# Discovery of Substituted (2-Aminooxazol-4-yl)Isoxazole-3-carboxylic Acids as Inhibitors of Bacterial Serine Acetyltransferase in the Quest for Novel Potential Antibacterial Adjuvants

**DOI:** 10.3390/ph14020174

**Published:** 2021-02-23

**Authors:** Joana Magalhães, Nina Franko, Samanta Raboni, Giannamaria Annunziato, Päivi Tammela, Agostino Bruno, Stefano Bettati, Stefano Armao, Costanza Spadini, Clotilde Silvia Cabassi, Andrea Mozzarelli, Marco Pieroni, Barbara Campanini, Gabriele Costantino

**Affiliations:** 1 P4T Group, Department of Food and Drug, University of Parma, 43124 Parma, Italy; joanarpmagalhaes@gmail.com (J.M.); giannamaria.annunziato@unipr.it (G.A.); agostino84@gmail.com (A.B.); gabriele.costantino@unipr.it (G.C.); 2 Laboratory of Biochemistry and Molecular Biology, Department of Food and Drug, University of Parma, 43124 Parma, Italy; nina.franko@studenti.unipr.it (N.F.); samanta.raboni@unipr.it (S.R.); Stefano.armao@unipr.it (S.A.); andrea.mozzarelli@unipr.it (A.M.); barbara.campanini@unipr.it (B.C.); 3 Institute of Biophysics, CNR, 56124 Pisa, Italy; stefano.bettati@unipr.it; 4 Centro Interdipartimentale Misure (CIM) ‘G. Casnati’, University of Parma, 43124 Parma, Italy; 5 Drug Research Program, Division of Pharmaceutical Biosciences, Faculty of Pharmacy, University of Helsinki, P.O. Box 56 (Viikinkaari 5 E), FI-00014 Helsinki, Finland; paivi.tammela@helsinki.fi; 6 Department of Medicine and Surgery, University of Parma, Via Volturno, 39, 43125 Parma, Italy; 7 National Institute of Biostructures and Biosystems, 00136 Rome, Italy; 8 Operative Unit of Animals Infectious Diseases, Department of Veterinary Science, University of Parma, Via del Taglio 10, 43126 Parma, Italy; Costanza.spadini@unipr.it (C.S.); clotildesilvia.cabassi@unipr.it (C.S.C.)

**Keywords:** adjuvant therapies, antimicrobial resistance, cysteine biosynthesis, non-essential targets, serine acetyltransferase

## Abstract

Many bacteria and actinomycetales use L-cysteine biosynthesis to increase their tolerance to antibacterial treatment and establish a long-lasting infection. In turn, this might lead to the onset of antimicrobial resistance that currently represents one of the most menacing threats to public health worldwide. The biosynthetic machinery required to synthesise L-cysteine is absent in mammals; therefore, its exploitation as a drug target is particularly promising. In this article, we report a series of inhibitors of *Salmonella thyphimurium* serine acetyltransferase (SAT), the enzyme that catalyzes the rate-limiting step of L-cysteine biosynthesis. The development of such inhibitors started with the virtual screening of an in-house library of compounds that led to the selection of seven structurally unrelated hit derivatives. A set of molecules structurally related to hit compound **5**, coming either from the original library or from medicinal chemistry efforts, were tested to determine a preliminary structure–activity relationship and, especially, to improve the inhibitory potency of the derivatives, that was indeed ameliorated by several folds compared to hit compound **5** Despite these progresses, at this stage, the most promising compound failed to interfere with bacterial growth when tested on a Gram-negative model organism, anticipating the need for further research efforts.

## 1. Introduction

Cysteine, which in mammals cannot be obtained via direct synthesis, is the main building block of many biomolecules and cofactors, such as S-adenosyl methionine, CoA, biotin, lipoic acid, and thiamin pyrophosphate, and it is the active portion of molecules with detoxifying properties such as glutathione and mycothiol. Besides being essential to all organisms, cysteine is particularly important for some facultative intracellular pathogens that must respond to nutrient starvation and/or oxidative stress occurring inside macrophages. In these challenging conditions, any interference with the adaptation strategies may lead to a reduction of their infectivity and to an increased susceptibility to antibacterial agents. Moreover, whereas cysteine biosynthetic enzymes are dispensable during growth in vitro or acute infection, they become indispensable during persistency [1,2]. In the last years, it has been demonstrated that reduction of cysteine biosynthesis or its suppression negatively affects bacterial fitness and virulence [2,3,4,5,6,7,8]. For instance, mutants bearing a deletion of cysteine biosynthetic enzymes were found to possess reduced virulence as in the case of cysH of *M. tuberculosis* [9] and cysI or cysK of *B. melitensis* [10], and/or an increased sensitivity to oxidative stress, like the mutant of cysI of *B. melitensis* [10]. One of the most intriguing aspects of cysteine biosynthesis is its role in the development of antibiotic resistance, as reported in the case of *S. typhimurium*, where an impaired oxidative stress response due to the genetic inhibition of cysteine biosynthesis caused a decrease in antibiotic resistance in both vegetative and swarm cell populations [11].

Altogether, these findings suggest that inhibition of cysteine biosynthesis might have the two-fold advantage of noticeably improving the action of many marketed antibacterial agents, counteracting their obsolescence, and preventing the onset of antimicrobial resistance. Since antimicrobial resistance (AMR) is regarded by many experts as the next-to-be healthcare scourge, the significance of the cysteine biosynthesis inhibition gains further therapeutic value. Experts estimate a loss of 10 million human lives every year due to antimicrobial resistance by 2050, unless innovative research actions are taken [12,13,14]. Along with the exploitation of novel druggable targets via proper drug discovery campaigns, a convenient strategy to counteract AMR would be to pursue unconventional approaches such as the use of adjuvant agents endowed with weak or absent antimicrobial activity that engage non-essential targets related to bacterial virulence and/or persistence after colonization of the host [15,16,17,18]. These targets are represented by those proteins which are dispensable during the normal bacterial life cycle, but become essential during host infection, as they mitigate the toxic action of antibiotics [18]. Among the many advantages in pursuing this strategy, adjuvants can suppress intrinsic resistance and therefore expand the activity spectrum of current antibiotics [19]. Similarly, for those antibiotics showing toxicity issues, adjuvants can allow for administration at lower doses, therefore diminishing the side effects [20]. In addition, such targets, being not essential for survival per se, are less likely to generate mutations and therefore induce resistance [21].

**Figure 1:**
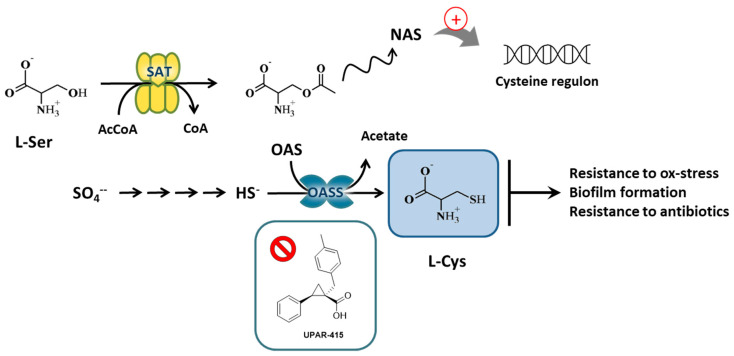
Schematic representation of the reductive sulfate assimilation pathway in Salmonella. Inorganic sulfate is transported inside the cell where it is reduced to bisulfide by a multi-step process. L-serine is activated by *O*-acetylation by SAT to give the product OAS, which spontaneously tautomerizes to NAS, the inducer of the cysteine regulon. A β-substitution reaction between OAS and HS^-^, catalyzed by OASS, leads to L-cysteine formation. This amino acid and the pathways of its biosynthesis influence many processes relevant to infection, like resistance to oxidative stress, biofilm formation and antibiotic resistance. UPAR415 is a competitive inhibitor of OASS.

**Figure 2:**
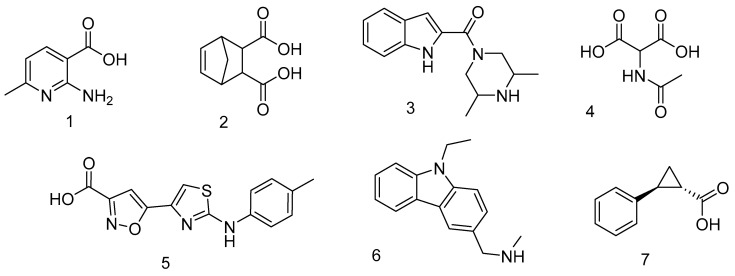
Compounds from the virtual screening.

In the final steps of cysteine biosynthesis, the transfer of the acetyl group from acetyl-CoA to serine, to form *O*-acetylserine (OAS), is catalyzed by serine acetyltransferase (SAT). In the following reaction, *O*-acetylserinesulfhydrylase (OASS) catalyzes the β-substitution of OAS with sulfide to give cysteine and acetate. Numerous efforts have been made by our research group [22,23,24,25] and others [26,27,28,29] toward the discovery of potent and selective OASS inhibitors, to be used either as antibacterials, especially towards *M. tuberculosis*, or adjuvants. This has led to the discovery and characterization of the most potent inhibitor of StOASS known so far; namely, **UPAR-415** (Figure 1) [30]. In spite of its potent activity in the biochemical assay, **UPAR-415** was not able to replicate a similar activity in bacterial cells, even though we have recently gained evidence that **UPAR-415** acts as a colistin adjuvant for pathogen infections (manuscript submitted). According to a preliminary analysis, the lack of efficacy in bacterial cells was due to permeability issues that prevented the molecule from reaching the intracellular targets. Indeed, poor permeability is one of the most common attrition elements in the antibacterial discovery process. Due to these results, it seemed reasonable to shift our focus also to the previous step of cysteine biosynthesis, catalyzed by SAT. Inhibition of SAT has been seldom investigated and the molecular tools reported so far exhibited only limited inhibitory properties towards the enzyme [26,31,32,33]. SAT catalyzes the transfer of the acetyl group of acetyl-CoA to the hydroxyl group of L-serine to generate OAS, the activated form of L-Ser. Compared to inhibition of OASS, the inhibition of SAT is expected not only to exert down-stream cysteine reduction, but also to inhibit accumulation of OAS that induces the biosynthetic operon upon conversion to *N*-acetylserine (NAS) [34]. From the structural point of view, SAT is a dimer of trimers, with an α-helical N-terminal domain and a carboxyl-terminal left-handed α-helix domain ending with a flexible, disordered C-terminal sequence that binds OASS-A at the active site and competitively inhibits it [35,36]. Our first attempt to deliver an SAT inhibitor relied on the virtual screening of a focused commercial library of chemical compounds [37]. This strategy was inspired by previous works where similar approaches were carried out obtaining encouraging results [27,33]. In our first attempt, the virtual screening of three ChemDiv focused libraries containing 91,243 compounds was performed to identify six compounds displaying an IC_50_ < 100 μM via an indirect assay using Ellman’s reagent [37]. In addition, one of the compounds was found to have measurable growth inhibitory effects against *E. coli*. We followed a similar approach to virtually assay an in-house library of chemical compounds that were initially designed to inhibit molecular targets other than SAT. Over the recent years, drug or chemical libraries repurposing is emerging as an extremely fruitful strategy to inspire drug discovery, and both big pharmaceutical companies and academic institutions screen in-house chemical libraries of compounds for purposes other than those for which they were initially conceived. This approach has the considerable advantage of potentially turning back to life molecules normally destined to oblivion, for which a biological/chemical characterization is often already available. Despite the smaller size of the library compared to the commercial one, the vs. was efficient in identifying a small set of promising hit compounds (Figure 2), for which the structure–activity relationships were investigated by means of both analogues already present in the library and synthesis of novel derivatives.

## 2. Results

### 2.1. Hit Compounds from Virtual Screening

Since the primary structure of SAT is highly conserved among bacteria, [38] the virtual screening of our in-house library was performed using the available crystal structures of *Ec*SAT (pdb code: 1T3D) and *Hi*SAT (pdb code: 1SSM), setting the experimental parameters as previously reported by us [37]. After this preliminary analysis, a total of 7 compounds were identified and selected to be evaluated on the recombinant enzyme from *S. typhimurium*. The enzyme used in these assays has a K*_m_*_,Ser_ = 1.07 ± 0.15 mM, a K_m,AcCoA_ = 0.17 ± 0.04 mM, k*cat* = 3813 ± 169 min^−1^ [37]. The assay conditions for the initial screening were set to improve the sensitivity of the assay towards both competitive and uncompetitive inhibitors, i.e., the concentration of both substrates was equal to K*_m_*.

**Figure 3:**
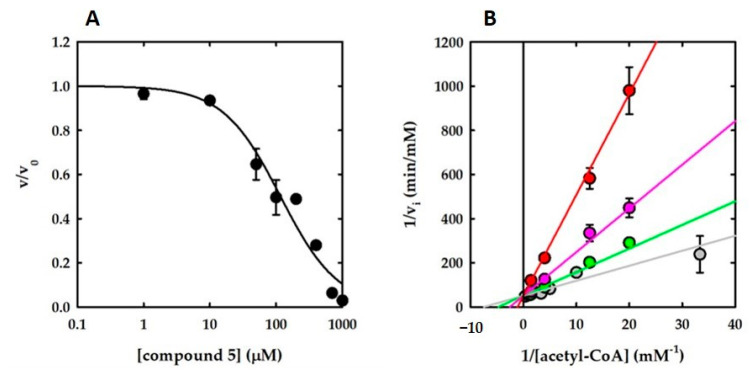
Inhibitory activity of compound **5**. Panel A: dependence of relative initial reaction rate on the concentration of compound **5**. The line through data points is the fitting to equation 1 with IC_50_ = 110 ± 0 μM. Panel B: dependence of the initial reaction rate on the concentration of acetylCoA in the absence of added inhibitors (grey dots) and in the presence of different concentrations of compound **5** (green dots 30 μM; pink dots 100 μM, red dots 300 μM). The series of lines through data points is the fitting to the linearised form of equation 2 with K_i_ = 64 ± 12 μM.

**Figure 4:**
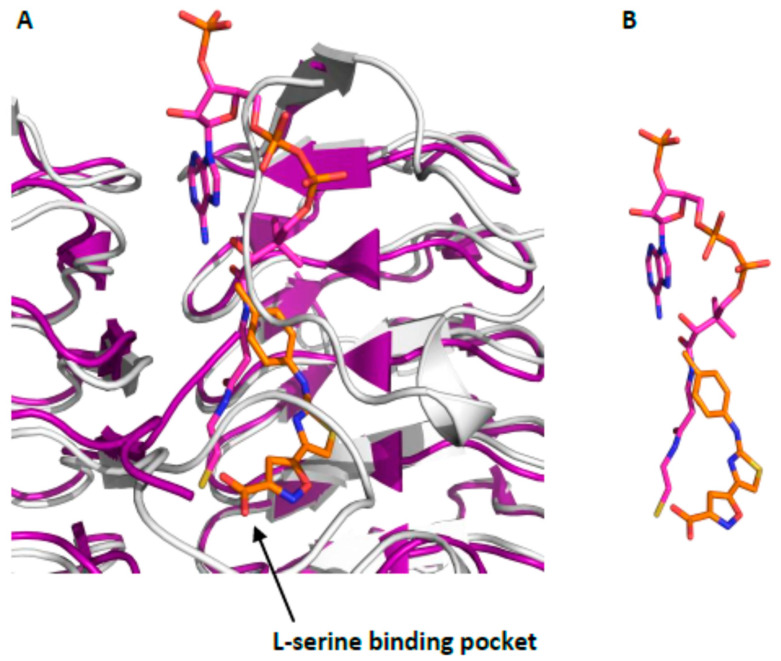
(**A**) docking pose of compound **5** in AcCoA pocket in EcSAT (alignment of structures with pdb code 1T3D and pdb code 1SSM). (**B**) Overlay of the docking pose of compound **5** and AcCoA structure.

Interestingly, six out of seven compounds are characterised by the presence of a carboxylic functional group, a structural requisite that seems to be crucial for properly interacting with the target. To rank the molecules and select a hit candidate for further expansion, the inhibitory potency of compounds **1–7** was assayed at a fixed concentration of 1mM (Table 1). Compounds **5** and **7** were found to inhibit the activity of the enzyme by more than 40% at the tested concentration, and they were therefore selected for IC_50_ determination. Consistent with the preliminary evaluation, compound **5** emerged as the most potent hit of the set displaying an IC_50_ of 110 ± 0 μM. On the other hand, compound **7** showed an IC_50_ > 2 mM. Further biochemical studies characterised compound **5** as a competitive inhibitor of the enzyme towards acetyl-CoA with a K*_i_* of 64 ± 12 μM (Figure 3). This was further corroborated by the computational investigation that predicted how the compound is accommodated in both L-serine and acetyl-CoA pockets. The L-shape allows partial filling of the acetyl-CoA cavity at the tail level, whereas the carboxylic acid functionality mimics the carboxylic acid group of the natural substrate, L-serine (Figure 4). This binding mode is compatible with a competitive displacement of acetyl-CoA, although interactions with other regions of the acetyl-CoA binding pocket are likely to happen. Compound **5** belongs to a series of antitubercular 2-aminothiazoles and it was initially designed and synthesised for anti-TB Structure-Activity Relationships (SAR) purposes [39]. Although many of these derivatives have remarkable activities toward *M. tuberculosis*, compound **5** was found to be inactive, and this is an advantage when a compound is repurposed to prevent the occurrence of off-target issues.

### 2.2. Hit Expansion

**Scheme 1:**
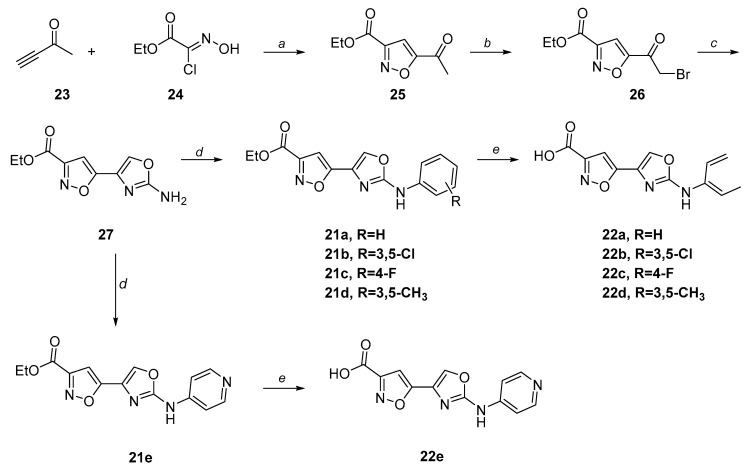
*^a^* Reagent and conditions: (*a*) triethylamine, tetrahydrofuran, rt; (*b*) N-Bromosuccinimide, *p*-toluenesulfonic acid, acetonitrile, reflux; (*c*) urea, dimethylformamide, MW (120 °C, 300 W), 3 min, 49%; (*d*) suitable bromobenzene derivative or 4-bromopyridine (**21e**), Sodium *t*-butoxide, X-Phos Pd G2, t-butanol, toluene, MW (130 °C, 300 W), 15 min, 8–35%; (*e*) LiOH, THF/H_2_O/MeOH 3:1:1, rt, 3 h, quantitative.

The plan to release a valuable hit compound against *St*SAT consisted of two steps. First, analogues of compound **5** already available in the in-house library were tested in order to build a preliminary body of SAR; then, based on the SAR obtained, the rational synthesis of additional analogues was carried out. A total of 13 in-house analogues of compound **5** were selected and tested for their capability to inhibit *St*SAT (Table 2). In particular, the carboxylic moiety was maintained either as acid or as its derivatives, namely esters and amides. In some cases, derivatives bearing an *N*-methylpyrazole in place of the isoxazole were considered for testing. Additionally, the effect of small electron withdrawing groups (EWGs) in the phenyl ring attached to the 2-aminothiazole was evaluated. The majority of the derivatives was found to be more potent than the hit compound **5**, with compounds **18** and **19**, where the 2-aminothiazole ring is replaced by a 2-aminooxazole, showing the most efficient inhibitory activity toward the enzyme. Inspired by these preliminary findings, in the second step synthetic efforts were devoted toward the synthesis of further analogues. In particular, a small set of analogues of the 2-aminooxazole derivatives **18** and **19** was designed and synthesised, taking advantage from a synthetic protocol that we have previously reported [40], which proved to be particularly versatile in the case of substituted 2-aminooxazoles (Scheme 1). The small set of derivatives consisted of 3-carboxylisoxazoles characterised by the presence of variously substituted aromatic and heteroaromatic rings attached at the nitrogen of the 2-aminooxazole (**21a**–**e**, **22a**–**e**). In this preliminary round of substitution, pyridine and benzene, unadorned or bearing electron-withdrawing or electron-donor groups, were used as substituents.

### 2.3. Chemistry

Scheme 1*^a^* Synthesis of compounds **21a–e** and **22a–e**.

The synthesis of the title compounds **21a–e** and **22a–e** was carried out via a synthetic protocol optimised in our laboratories relying on the condensation of the proper α-bromoacetophenones with urea followed by the Buchwald–Hartwig cross coupling of the 2-aminooxazole with an aryl halide. Dipolar cycloaddition between 3-butyn-2-one and ethyl 2-chloro-2-(hydroxyimino)acetate in diethyl ether and trietylamine (TEA) led to the synthesis of ethyl 5-acetyl-3-isoxazolecarboxylate **25**, that was efficiently brominated with N-bromosuccinimide (NBS) to yield derivative **26**. Reaction of **26** with urea in a microwave reactor at 120 °C in dimethylformamide led to the synthesis of building block **27**, which underwent Buchwald–Hartwig cross coupling with variously substituted bromobenzenes to afford the title compounds **21a-e**. Hydrolysis of the ethyl esters with LiOH in a mixture of THF/MeOH/H_2_O gave the corresponding acids **22a-e** in quantitative yields.

## 3. Discussion

### 3.1. Hints of Structure–Activity Relationships

In this research work, valuable hit compounds able to inhibit *St*SAT are reported through a virtual screening and subsequent hit optimization. Since compound **5** emerged as the most potent hit from the virtual screening, the first body of SAR was obtained by testing a collection of 13 structurally related compounds already available in the in-house library. The majority of the derivatives tested was found to be more affine to *St*SAT than the hit compound **5**. To confirm the importance of the carboxylic moiety, we first evaluated its abolition. Substitution of the isoxazole-3-carboxylic core with a pyridine led to loss of the affinity for the enzyme (**8**, IC_50_ = >400 μM). In general, the presence of the carboxylic functional group, either as a carboxylic acid (**16**, IC_50_ = 184 μM, **17**, IC_50_ = 21 μM, **18**, IC_50_ = 2.6 μM, **20**, IC_50_ = 11 μM) or its derivatives such as amides (**10**, IC_50_ = 9 μM, **11**, IC_50_ = 26 μM, **12**, IC_50_ = 60 μM, **13**, IC_50_ = 16.3 μM) and esters (**14**, IC_50_ = 10 μM, **15**, IC_50_ = 18 μM, **19**, IC_50_ = 7.3 μM), despite the different substitution pattern, seems to play a role in maintaining acceptable inhibitory activity, the only exception being represented by **9**, which shows promising activity despite devoid of the carboxylic functionality (**9**, IC_50_ = 12 μM). It might be hypothesised that the electronic pattern of carboxylic acid derivatives (i.e., acid, ester and amides) is favorable for the interaction with the enzyme. Among the amides, it seems that bulky substituents such as the phenyl ring and the adamantane attached at the nitrogen confer higher affinity than a methyl or unsubstituted amide (*cfr* **10** and **13** vs. **12** and **11**). When the isoxazole ring is replaced with a *N*-methylpyrazole (**16**, IC_50_ = 184 μM, **17**, IC_50_ = 21 μM), a reduction of the inhibitory activity is noticed compared to the isoxazole counterpart **5** and **20**. Among the isoxazole-3-carboxylic acid derivatives, introduction of electron withdrawing groups (EWGs) in the phenyl ring attached to the 2-aminothiazole seems to have beneficial effect on the affinity (**20**, IC_50_ = 11 μM vs. **5**, IC_50_ = 110 μM), although at this stage this consideration is quite speculative. An ester moiety can efficiently replace the carboxylic acid group (**14**, IC_50_ = 10 μM and **15**, IC_50_ = 18 μM), regardless the nature of the substituent attached at the 2-aminothiazole. For compound **14**, it must be specified that the IC_50_ could be only estimated due to some solubility issue. The difference in the activity for compounds **5** and **14** cannot be easily explained, unless permeability is brought up. However, this does not seem to be the case. The synthesis of additional analogues can be of help in explaining this discrepancy. Replacement of the 2-aminothiazole moiety by a 2-aminooxazole led to the most potent derivatives of the series (**18**, IC_50_ = 2.6 μM and **19**, IC_50_ = 7.3 μM), with a >40-fold improvement in the affinity compared to the 2-aminothiazole counterpart (**18**, IC_50_ = 2.6 μM vs. **5**, IC_50_ = 110 μM). These findings prompted us to synthesise a small set of derivatives for further SAR refinement, evaluating the presence of small functional groups on the phenyl ring attached to the 2-aminooxazole nitrogen and investigating whether the ethyl ester or the acid had the best affinity for the enzyme. Biochemical evaluation of compounds **21a–e** and **22a–e** on *St*SAT showed that, in general, the SAR obtained with these small modifications was quite flat (Table 3). Both the carboxylic acid and its ethyl ester derivatives are equally able to confer adequate inhibitory activity, (compounds **21a–e vs.** compounds **22a–e**). Ester derivatives can be considered slightly more efficient than the acid counterparts, even though compound **22a**, an acid, was found to be the most active derivative obtained from this study. Overall, compound **22a** and the other acid derivatives might be considered valuable hit compounds for further investigation. This might be due to the fact that acids are generally metabolically more stable than the corresponding esters, which can undergo hydrolysis. Additionally, acid derivatives have no intrinsic antitubercular or antibacterial activity [39], an important requirement to avoid off-target effects during the repurposing process. Introduction of small EWGs (compounds **21b**, **21c**, **22b**, **22c**) or electron-donor groups (compound **22d**) at different positions of the phenyl ring does not interfere significantly with the affinity of the molecules for *St*SAT, although the most interesting results are obtained when the phenyl ring is not substituted (compounds **21a** and **22a**).

Having in mind that compound **5** is a competitive inhibitor of acetyl CoA predicted by the computational studies to be accommodated in both pockets of acetyl CoA and L-serine, it is possible that the structure we are currently exploring is only occupying a part of the pocket and the substitutions we are performing are all tolerable due to the empty space in the cavity.

### 3.2. Stability of the Isoxazole-Oxazole Nucleus

Upon noticing changes in the appearance of the DMSO solution over time at room temperature, we deemed it of interest to investigate the chemical stability of the compounds. Therefore, in order to evaluate it, ^1^H NMR analysis at different time points (1 h, 2.5 h, and 20 h) of the DMSO solution (5 mg in 500 μL of DMSO-d6) was performed (Supporting Information). Since the time required to observe changes in the colour of the solution was dependent of the substituent at position R2 of series **22**, to establish the effect of the substituent on the overall stability of the compound, analogue **22a** (R2 = phenyl) which contains the non-substituted phenyl group, analogue **22b** (R2 = 3,5-dichlorophenyl), substituted with an EWG and analogue **22d** (R2 = 3,5-dimethylphenyl) substituted with an electron donating group (EDG) were selected to perform the chemical stability studies. The stability of the compounds increases in the following order: **22b** (stability in DMSO < 2.5 h) < **22a** (stability in DMSO < 20 h) < **22d** (stability in DMSO < 7 days). Thus, it is possible to conclude that EWGs like the simple phenyl or the phenyl replaced with two chlorines produce fewer stable molecules. On the other hand, the phenyl replaced with EDGs contributes to the overall stability of the molecule. It is hypothesised that the mild oxidative properties of DMSO are able to interfere with the oxazole scaffold, which leads to the opening of the ring and then full degradation of the compound in study [41].

### 3.3. Antibacterial Activity

Considering the stability issues above described, compound **22a** did not undergo biological evaluation. Rather, compound **22d**, which is only two-fold less active that **22a** in the biochemical assay, was tested for its MIC against *E. coli* both in standard Mueller Hinton broth (MHB) and in Middlebrook 9 (M9), which is deprived of cysteine [42]. Unfortunately, in both these conditions, no antibacterial activity could be measured at concentration ≤128 μM on two bacterial strains, namely *E. coli* ATCC25922 and *S. Typhimurium* ATCC14028. A plausible cause for the lack of activity might be the poor permeability of this compound through the bacterial outer membrane and cell wall, which is one of the most common issues related to antibacterial drug discovery, especially toward Gram-negative species.

## 4. Materials and Methods

### 4.1. Enzyme Preparation and Activity Assay

SAT from *Salmonella enterica* serovar Typhimurium was expressed as trxA fusion protein as previously described [37] and was more than 95% pure based on SDS-PAGE analysis. The enzyme activity was measured by a spectrophotometric indirect assay that exploits the signal at 412 nm generated by 5,5’-Dithiobis(2-nitrobenzoic acid (Ellmn’s reagent) reduction by the reaction product coenzyme A [37]. A total of 5% DMSO was present in all the assays and was proven not to perturb enzyme activity (data not shown). The concentration of substrates was set equal to *K_m_* in order to improve the sensitivity of the assay for both competitive and uncompetitive mechanism of inhibition [43]. The dependence of the relative reaction rate on the concentration of compounds was fitted to Equation (1) to calculate the *IC*_50_:

$$\frac{v_{i}}{v_{0}}=\frac{1}{1+\frac{\left[I\right]}{IC_{50}}}$$

The inhibition mechanism was determined by fitting the dependence of the initial reaction rate on the concentration of acetyl CoA (at fixed, 1 mM L-Ser concentration) in the presence of different concentrations of inhibitor ($\frac{1}{3}IC_{50}\geq \left[inhibitor\right]\leq 3IC_{50}$) to the equation for competitive, uncompetitive and non-competitive mechanisms [43]. The equation that fits a competitive mechanism (Equation (2)) was used to calculate *K_i_* for compound 5:

$$v_{0}=\frac{V_{max}\cdot \left[acetyl-CoA\right]}{\left[acetyl-CoA\right]+\left(K_{m}\cdot \alpha \right)}$$

where $\alpha =1+\left(\frac{\left[comp5\right]}{K_{i}}\right)$.

### 4.2. Chemistry

All the reagents were purchased from Sigma-Aldrich, Alfa-Aesar, and Enamine at reagent purity and, unless otherwise noted, were used without any further purification. Dry solvents used in the reactions were obtained by distillation of technical grade materials over appropriate dehydrating agents. Microwave reactions were performed using CEM microwave synthesizer Discover model. Reactions were monitored by thin layer chromatography on silica gel coated aluminium foils (silica gel on Al foils, SUPELCO Analytical, Sigma-Aldrich) at both 254 and 365 nm wavelengths. When indicated, intermediates and final products were purified through silica gel flash chromatography (silica gel, 0.040–0.063 mm), using appropriate solvent mixtures. ^1^H NMR and ^13^C NMR spectra were recorded on a BRUKER AVANCE spectrometer at 300, 400, and 100 MHz, respectively, with TMS as internal standard. ^1^H NMR spectra are reported in this order: multiplicity and number of protons. Standard abbreviation indicating the multiplicity was used as follows: s = singlet, d = doublet, dd = doublet of doublets, t = triplet, q = quadruplet, m = multiplet and br = broad signal. HPLC/MS experiments were performed with HPLC instrumentation (Agilent 1100 series, equipped with a Waters Symmetry C18, 3.5 μm, 4.6 mm × 75 mm column) and MS instrumentation (Applied Biosystem/MDS SCIEX, with API 150EX ion source). HRMS experiments were performed with LTQ ORBITRAP XL THERMO. All compounds were tested as 95% purity samples or higher (by HPLC/MS).

Analytical data for compounds **8‒20**, **23‒27** have been already published [39,40,44].

Ethyl 5-acetylisoxazole-3-carboxylate (**25**): To a solution of butynone 23 (2.3 mL, 29 mmol) and ethyl-2-chloro-2-(hydroxyimino)acetate 24 (4.39 g, 29 mmol) in THF (37.5 mL) a solution of triethylamine (4 mL, 29 mmol) in THF (10 mL) was added dropwise. Reaction mixture was allowed to stir at room temperature for 3h and then volatiles were evaporated. Extractions with ethyl acetate were performed (3 × 50 mL) and the combined organic layers were dried over anhydrous sodium sulfate, filtered and concentrated under reduced pressure to be purified by flash column chromatography on silica gel using ethyl acetate/petroleum ether (7:93). The product was obtained as a white powder in 50% yield. ^1^H NMR (400 MHz, CDCl3) δ 7.26 (s, *J* = 2.0 Hz, 1H), 4.47 (q, *J* = 7.1 Hz, 2H), 2.65 (s, 1H), 1.43 (t, *J* = 7.1 Hz, 3H).

Ethyl 5-(2-bromoacetyl)isoxazole-3-carboxylate (**26**). To a solution of compound **25** (2.65 g, 14.47 mmol) in acetonitrile (26.8 mL) stirring on an ice bath, *p*-toluenesulfonic acid monohydrate (2.49 g, 14.47 mmol) and N-bromosuccinimide (2.58 g, 14.47 mmol) were added portion-wise. Reaction mixture was stirred at reflux for 5 h. After volatiles were evaporated and reaction mixture was purified by flash column chromatography on silica gel using a gradient of ethyl acetate in petroleum ether. The product was obtained as a yellow powder in 58% yield. ^1^H NMR (300 MHz, DMSO) δ 7.89 (s, 1H), 4.87 (s, 2H), 4.41 (dt, *J* = 12.4, 4.5 Hz, 2H), 1.34 (t, *J* = 7.1 Hz, 3H).

Ethyl 5-(2-aminooxazol-4-yl)isoxazole-3-carboxylate (**27**). A mixture of compound **26** (315 mg, 1.2 mmol) and urea (721 mg, 12 mmol) were solubilised in anhydrous dimethylformamide (3.14 mL) and stirred at reflux for 2 h, and then cooled until room temperature. After, a solution of LiCl 5% in water (32 mL) was added to the reaction mixture and extractions with ethyl acetate were performed (4 **×** 10 mL). The reaction mixture was then quenched with water and the mixture extracted with ethyl acetate (3 × 10 mL). The organic layers were washed with water, brine and dried over Na_2_SO_4_. After filtration, the solvent was removed in vacuo and the crude material was purified by flash column chromatography. The product was obtained as a yellow powder in 77% yield. ^1^H NMR (300 MHz, DMSO) δ 8.15 (s, 1H), 7.07 (s, 2H), 6.89 (s, 1H), 4.37 (dd, *J* = 13.8, 6.8 Hz, 2H), 1.32 (t, *J* = 6.9 Hz, 3H).

General procedure for the Buchwald–Hartwig reaction. Synthesis of compounds **21a‒e**. In a 10 mL test tube for microwave, a solution of the proper 2-aminooxazole (1 equiv), the suitable aryl halide (0.5 equiv), the base (1 equiv) in a mixture of anhydrous toluene (2.5 mL/mmol) and *t*-butanol (0.5 mL/mmol) was stirred under argon flux for 15 min. After this time, the suitable catalyst (0.1 equiv) was added and the reaction mixture was irradiated in a microwave reactor after setting the parameters as follows: temperature = 130 °C, time = 15 min, pressure = 250 psi, power = 300 W, power max = off. The reaction was monitored by TLC. After the complete consumption of the starting material, as revealed by TLC (95:5 dichloromethane/methanol), water was added, and the mixture extracted with ethyl acetate (3 × 10 mL). The organic layers were washed with water, brine and dried over Na_2_SO_4_. After filtration, the solvent was removed in vacuo and the crude material was purified by flash column chromatography.

Ethyl 5-(2-(phenylamino)oxazol-4-yl)isoxazole-3-carboxylate (**21a**). Purification by flash column chromatography using ethyl acetate/petroleum ether (1:9) afforded the product as a yellow solid in 24% yield. ^1^H NMR (400 MHz, Acetone) δ 9.41 (s, 1H), 8.17 (s, 1H), 7.76 (d, *J* = 7.9 Hz, 2H), 7.36 (t, *J* = 7.9 Hz, 2H), 7.12–6.75 (m, 2H), 4.43 (q, *J* = 7.1 Hz, 2H), 1.39 (t, *J* = 7.1 Hz, 3H). ^13^C-NMR (100.6 MHz; Acetone): δ = 20.8; 62.4; 101.5; 117.4; 122.4; 129.8; 132.7; 136.9; 156.5; 156.6; 158.1; 159.3; 165.1. HRMS (ESI) calculated for C_15_H_13_N_3_O_4_ [M + H]^+^, 300.0906 found 300,1112.

Ethyl 5-(2-((3,5-dichlorophenyl)amino)oxazol-4-yl)isoxazole-3-carboxylate (**21b**). Purification by flash column chromatography using ethyl acetate/petroleum ether (1:9) afforded the product as an orange powder in 30% yield. ^1^H NMR (300 MHz, CDCl_3_) δ 7.82 (s, 1H), 7.51 (d, *J* = 1.6 Hz, 2H), 7.07 (s, 1H), 7.02 (s, 2H), 4.48 (q, *J* = 7.1 Hz, 2H), 1.45 (t, *J* = 7.1 Hz, 3H). ^13^C-NMR (100.6 MHz; CDCl_3_): δ = 18.5; 62.1; 101.3; 108.8; 119.8; 120.2; 128.0; 139.9; 150.1; 154.8; 156.8; 158.2; 160.5. HRMS (ESI) calculated for C_15_H_11_Cl_2_N_3_O_4_ [M + H]^+^, 368.0126 found 368.0520.

Ethyl 5-(2-((4-fluorophenyl)amino)oxazol-4-yl)isoxazole-3-carboxylate (**21c**). Purification by flash column chromatography using ethyl acetate/petroleum ether (1:9) afforded the product as a slightly orange powder in 8% yield. ^1^H NMR (300 MHz, CDCl_3_) δ 7.77 (s, 1H), 7.51 (dd, *J* = 8.4, 4.2 Hz, 2H), 7.07 (t, *J* = 8.5 Hz, 2H), 6.98 (s, 1H), 6.92 (s, 1H), 4.47 (q, *J* = 7.1 Hz, 2H), 1.44 (t, *J* = 7.1 Hz, 3H). ^13^C-NMR (100.6 MHz; CDCl_3_): δ = 18.9; 62; 100.8; 108.2; 121.0; 121.2; 127.8; 139.9; 150.1, 154.8; 157.3; 159.2; 160.8. HRMS (ESI) calculated for C_15_H_12_FN_3_O_4_ [M + H]^+^, 318.0811 found 318.0822.

Ethyl 5-(2-((3,5-dimethylphenyl)amino)oxazol-4-yl)isoxazole-3-carboxylate (**21d**). Purification by flash column chromatography using ethyl acetate/petroleum ether (1:9) afforded the desired product as a yellow powder in 35% yield. ^1^H NMR (300 MHz, DMSO-d_6_) δ 10.28 (s, 1H), 8.42 (s, 1H), 7.25 (s, 2H), 7.10 (s, 1H), 6.63 (s, 1H), 4.39 (q, *J* = 7.1 Hz, 2H), 2.26 (s, 6H), 1.34 (t, *J* = 7.1 Hz, 3H). ^13^C-NMR (100.6 MHz; DMSO-d_6_): δ = 18.8; 21.8, 21.9, 61.8; 100.8; 112.8; 121.4; 126.2; 138.8; 140.1; 150.8, 155; 156.8; 159.2; 160.8. HRMS (ESI) calculated for C_17_H_17_N_3_O_4_ [M + H]^+^, 328.1219 found 328.1401.

Ethyl 5-(2-(pyridin-4-ylamino)oxazol-4-yl)isoxazole-3-carboxylate (**21e**). Purification by flash column chromatography using ethyl acetate/petroleum ether (1:1→100%) afforded the product as a yellow powder in 15% yield. ^1^H NMR (400 MHz, Acetone) δ 8.47 (d, *J* = 6.2 Hz, 2H), 8.27 (s, 1H), 7.73 (d, *J* = 6.3 Hz, 2H), 7.07 (s, 1H), 4.44 (q, *J* = 7.1 Hz, 2H), 1.40 (t, *J* = 7.1 Hz, 3H). ^13^C-NMR (100.6 MHz; Acetone): δ = 21; 62.4; 101.3; 109,0; 125.4; 139.2; 150.1, 155.6; 156.6; 158.1; 159.3; 160.5. HRMS (ESI) calculated for C_14_H_12_N_4_O_4_ [M + H]^+^, 301.0858 found 301,0999.

General procedure for the hydrolysis. Synthesis of compounds **22a‒e**. The suitable ester (1 equiv) and LiOH·H_2_O (4 equiv) were dissolved in a mixture of THF/MeOH/H_2_O (3:1:1, 1 mL/mmol) and stirred at room temperature until consumption of the starting material as indicated by TLC (7:3 petroleum ether/ethyl acetate, then 9:1 Dichloromethane/methanol). The reaction mixture is then evaporated under reduced pressure, and the crude obtained is taken up with H_2_O, acidified with 2N HCl and extracted with ethyl acetate (3 × 10 mL). After evaporation of the solvent, the desired acid derivatives are obtained in sufficient purity.

5-(2-(phenylamino)oxazol-4-yl)isoxazole-3-carboxylic acid (**22a**). Yellow powder (37% yield); ^1^H NMR (400 MHz, DMSO-d_6_) δ 10.45 (s, 1H), 9.47 (s, 1H) 8.42 (s, 1H), 7.66 (d, *J* = 7.8 Hz, 2H), 7.34 (t, *J* = 7.9 Hz, 2H), 7.00 (dd, *J* = 15.0, 7.6 Hz, 2H). ^13^C NMR (101 MHz, DMSO-d_6_) δ 164.5; 160.6; 157.4; 138.8; 132.1; 129.0; 128.9; 121.6; 116.8; 101.2. HRMS (ESI) calculated for C_13_H_9_N_3_O_4_ [M + H]^+^, 272.0593 found 272.0606.

5-(2-((3,5-dichlorophenyl)amino)oxazol-4-yl)isoxazole-3-carboxylic acid (**22b**). Light yellow powder (60% yield); ^1^H NMR (400 MHz, DMSO-d_6_) δ 11.01 (s, 1H), 8.49 (s, 1H), 7.72 (d, *J* = 1.8 Hz, 2H), 7.19 (t, *J* = 1.8 Hz, 1H), 7.03 (s, 1H). ^13^C NMR (101 MHz, DMSO-d_6_) δ 160.4; 156.2; 141.0; 134.2; 132.5; 127.9; 124.0; 120.5; 114.7; 101.2. HRMS (ESI) calculated for C_13_H_7_Cl_2_N_3_O_4_ [M + H]^+^, 339.9814 found 339.9986.

5-(2-((4-fluorophenyl)amino)oxazol-4-yl)isoxazole-3-carboxylic acid (**22c**). Yellow powder (81% yield); ^1^H NMR (400 MHz, Acetone) δ 9.48 (s, 1H), 8.18 (s, 1H), 7.85–7.77 (m, 2H), 7.19–7.11 (m, 2H), 7.01 (s, 1H). ^13^C NMR (101 MHz, Acetone) δ 166.0; 161.0; 160.1; 158.6; 158.0; 136.3; 132.3; 129.8; 119.6; 119.5; 116.4; 116.2; 102.2. HRMS (ESI) calculated for C_13_H_8_FN_3_O_4_ [M + H]^+^, 290.0499 found 290.0509.

5-(2-((3,5-dimethylphenyl)amino)oxazol-4-yl)isoxazole-3-carboxylic acid (**22d**). Yellow powder (68% yield); ^1^H NMR (400 MHz, DMSO-d_6_) δ 10.28 (s, 1H), 8.41 (s, 1H), 7.26 (s, 2H), 7.04 (s, 1H), 6.64 (s, 1H), 2.27 (s, 6H). ^13^C NMR (101 MHz, DMSO-d_6_) δ 165.0; 161.0; 157.9; 157.7; 139.1; 138.4; 132.4; 128.5; 123.8; 115.0; 101.6; 21.6. HRMS (ESI) calculated for C_15_H_13_N_3_O_4_ [M + H]^+^, 300.0906 found 300.1002.

5-(2-(pyridin-4-ylamino)oxazol-4-yl)isoxazole-3-carboxylic acid (**22e**). Yellow powder (63% yield); ^1^H NMR (300 MHz, DMSO-d_6_) δ 12.58 (s, 1H), 8.71 (s, 1H), 8.66 (d, *J* = 7.2 Hz, 2H), 8.12 (d, *J* = 5.6 Hz, 2H), 7.20 (s, 1H). ^13^C NMR (101 MHz, DMSO-d_6_) δ 163.6; 160.5; 157.5; 155.0; 143.0; 134.2; 128.2; 112.2; 102.0. HRMS (ESI) calculated for C_12_H_8_N_4_O_4_ [M + H]^+^, 273.0545 found 273.0628.

### 4.3. Biology

All reference bacterial strains (4–5 morphologically similar colonies) were inoculated in MHB e M9 and incubated at 37 °C, in agitation at 240 rpm until log phase was reached. The bacterial suspension was then centrifuged 20 min at 2000 rpm and 4 °C, then the pellet was resuspended in MHB and M9 media. The turbidity of the bacterial suspension was immediately measured and adjusted by spectrophotometry. At 600 nm, the OD range 0.08–0.13 was considered to correspond to a bacterial concentration of 10^8^ CFU/mL [45]. 100 µL of the obtained suspension were further diluted in 9.9 mL of MHB and M9 media to obtain a final bacterial concentration of 10^6^ CFU/mL. An amount of 50 µl of the bacterial suspension were inoculated within 30 min into the wells of a microtiter plate to obtain 5 × 10^5^ CFU/mL, according with CLSI guidelines [45]. Antimicrobial activity of **22a**, **22b**, **22d** was assessed by Minimal Inhibitory Concentration (MIC) assay. Briefly, in a microtiter plate 10 wells were inoculated with 49 µL of M9 or MHB media. 1 μL of each compounds originally diluted in DMSO was then added to each well, thus testing concentrations ranging from 128 μg/mL to 0.25 μg/mL with a final concentration of 5 × 105 CFU/mL of bacterial suspension in a volume of 100 μL. Growth and sterility controls were performed. For each bacterial strain three independent experiments, with three replicates each, were performed.

## 5. Conclusions

Through an in-house library virtual screening combined with a medicinal chemistry campaign, we were able to identify the most potent inhibitor of *St*SAT known so far. This compound was however found to show instability issues when solubilised in DMSO; therefore, other derivatives, endowed with higher stability, were used for the following cellular assay. The most promising compound of the series (**22d**) was tested against *E. coli* in a minimum medium characterised by the absence of cysteine. Despite the remarkable activity in the biochemical assay, the compound was not able to interfere with the bacterial growth, likely for its inability to cross the bacterial membrane. This investigation allowed us to delineate a preliminary SAR, although the synthesis of further analogues is currently ongoing to further expand the set of structural information and, more in particular, to afford molecules with ameliorated permeability.

## Figures and Tables

**Table 1:** Inhibition of SAT by compounds identified in the virtual screening.

Cpd	% Inhibition at 1 mM	IC_50_ (μM)	Ki (μM)	Mechanism
1	19.4 ± 1	ND *^a^*	ND	
2	34.9 ± 6	ND	ND	
3	31.4 ± 5	ND	ND	
4	18.7 ± 6	ND	ND	
5	98.6 ± 0	110 ± 0	64 ± 12	Competitive with AcCoA
6	23.6 ± 0	ND	ND	
7	42 ± 5	>2 mM	ND	

*^a^* ND = Not Determined.

**Table 2:** *St*SAT Inhibitory activity of compound **5** analogues.

Cpd	Structure	IC_50_ (μM)	Cpd	Structure	IC_50_ (μM)
**8**	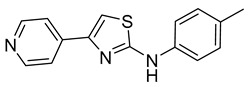	>400	**15**	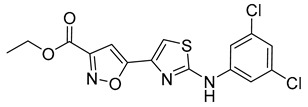	18 ± 2
**9**	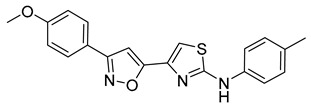	12 ± 2	**16**	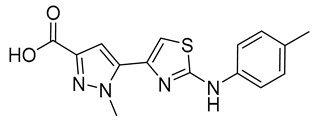	184 ± 13
**10**	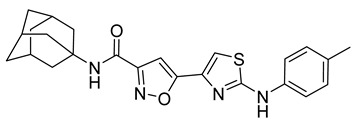	9 ± 3	**17**	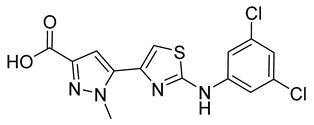	21 ± 5
**11**	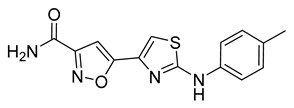	26 ± 4	**18**	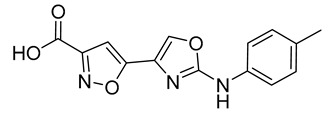	1.0 ± 0.2
**12**	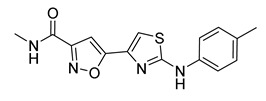	> 60 *	**19**	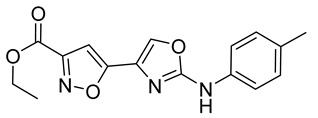	7 ± 2
**13**	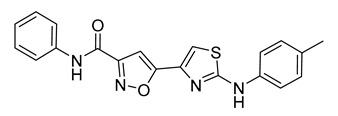	16 ± 3	**20**	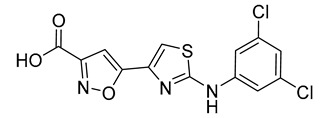	11 ± 1
14	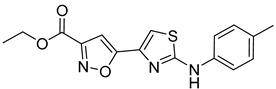	~10 *			

* The IC_50_ could only be estimated due to solubility issues of the compound.

**Table 3:** *St*SAT Inhibitory activity of synthesised compounds **21a–c, 21e** and **22 a–e.**

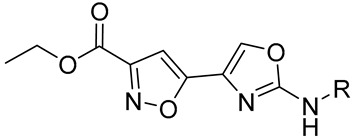			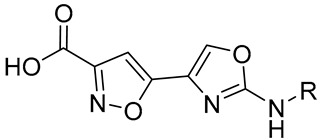		
Cmpd	R	*St*SAT IC_50_ (μM)	Cmpd	R	*St*SAT IC_50_ (μM)
**21a**	Phenyl	2.68 ± 0.27	**22a**	Phenyl	1.54 ± 0.33
**21b**	3,5-Dichlorophenyl	2.52 ± 0.03	**22b**	3,5-Dichlorophenyl	8.03 ± 0.18
**21c**	4-Fluorophenyl	3.04 ± 0.37	**22c**	4-Fluorophenyl	2.51 ± 0.34
			**22d**	3,5-Dimethylphenyl	4.24 ±0.11
**21e**	Pyridine	3.95 ± 0.65	**22e**	Pyridine	12.02 ± 1.25

## Data Availability

The data presented in this study are available in this article and related Supplementary Material.

## Supplementary Materials

The following are available online at https://www.mdpi.com/1424-8247/14/2/174/s1.
