# Supplementary material for: Discovery of Substituted (2-Aminooxazol-4-yl)Isoxazole-3-carboxylic Acids as Inhibitors of Bacterial Serine Acetyltransferase in the Quest for Novel Potential Antibacterial Adjuvants
Source: Pharmaceuticals (Basel). 2021 Feb 23;14(2):174. doi: 10.3390/ph14020174 (PMC7931047; doi:10.3390/ph14020174)

Discovery of substituted (2-aminooxazol-4-yl)isoxazole-3-carboxylic acids as inhibitors of bacterial serine acetyltransferase in the quest for novel potential antibacterial adjuvants

Joana Magalhães[1], Nina Franko[2], Samanta Raboni[2,7], Giannamaria Annunziato[1,8], Päivi Tammela[3], Agostino Bruno,[1] Stefano Bettati[4,6,7], Stefano Armao[2], Costanza Spadini[5], Clotilde Silvia Cabassi[5], Andrea Mozzarelli[2,6,7], Marco Pieroni[1,8,*], Barbara Campanini[2], and Gabriele Costantino[1,8]

1 P4T group, Department of Food and Drug, University of Parma, 43124 Parma, Italy;
2 Laboratory of Biochemistry and Molecular Biology, Department of Food and Drug, University of Parma, 43124 Parma, Italy;
3 Drug Research Program, Division of Pharmaceutical Biosciences, Faculty of Pharmacy, University of Helsinki, P.O. Box 56 (Viikinkaari 5 E), Helsinki, FI-00014, Finland;
4 Department of Medicine and Surgery, University of Parma, Via Volturno, 39, 43125 Parma;
5 Operative Unit of Animals Infectious Diseases, Department of Veterinary Science, University of Parma, via del Taglio 10, 43126 Parma, Italy.
6 National Institute of Biostructures and Biosystems, Rome, Italy;
7 Institute of Biophysics, CNR, Pisa, Italy;
8 Centro Interdipartimentale Misure (CIM) 'G. Casnati', University of Parma, Parma, Italy;
* Correspondence: marco.pieroni@unipr.it; Tel. +39-0521-905054

Contents

1. H-NMR of compounds 22a, 22c, 22e at different time points S2

JRM125_t0
JRM125_t0 DMSO 06/03/18

^{1}H NMR (400 MHz, DMSO) δ 10.28 (s, 1H), 8.40 (s, 1H), 7.25 (s, 2H), 7.03 (s, 1H), 6.64 (s, 1H), 2.26 (s, 6H).

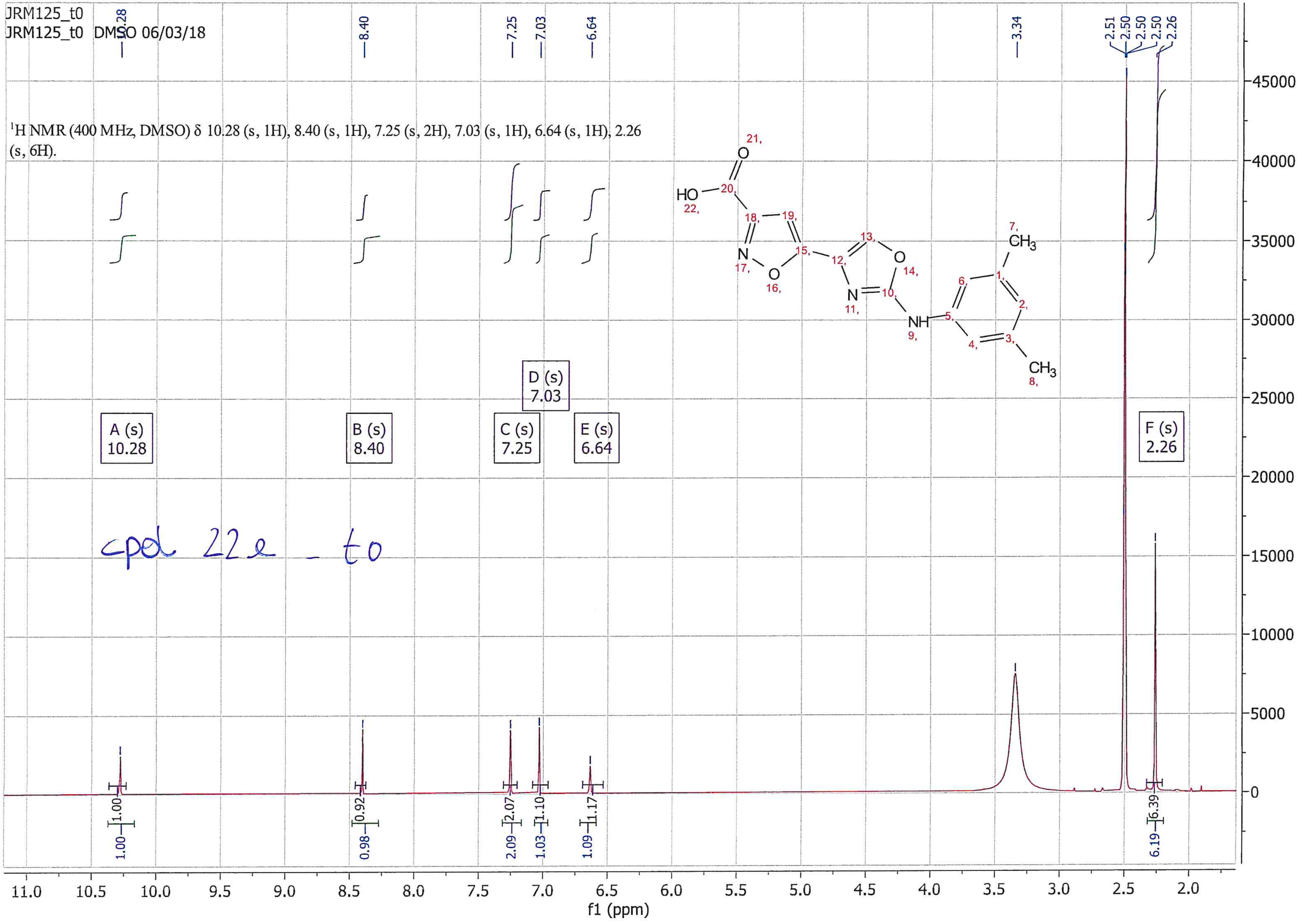

cpd 22e - t0

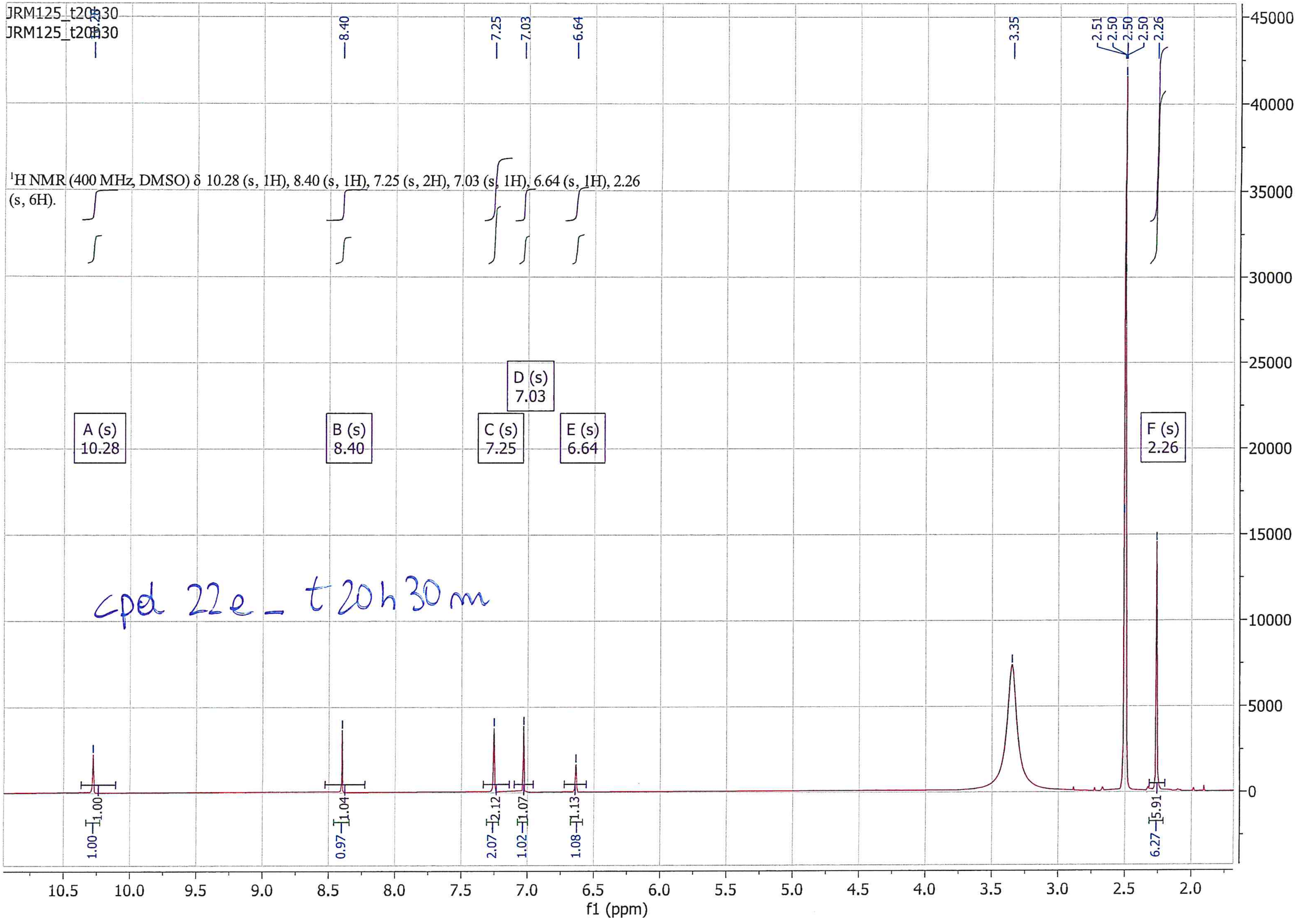
JRM125_t20h30
JRM125_t20h30
10.28
8.40
7.25
7.03
6.64
3.35
2.51
2.50
2.50
2.50
2.26
^{1}H NMR (400 MHz, DMSO) δ 10.28 (s, 1H), 8.40 (s, 1H), 7.25 (s, 2H), 7.03 (s, 1H), 6.64 (s, 1H), 2.26 (s, 6H).
A (s) 10.28
B (s) 8.40
C (s) 7.25
D (s) 7.03
E (s) 6.64
F (s) 2.26
cpd 22e - t 20h30m
1.00
1.00
0.97
1.04
2.07
2.12
1.02
1.07
1.08
1.13
6.27
5.91
f1 (ppm)

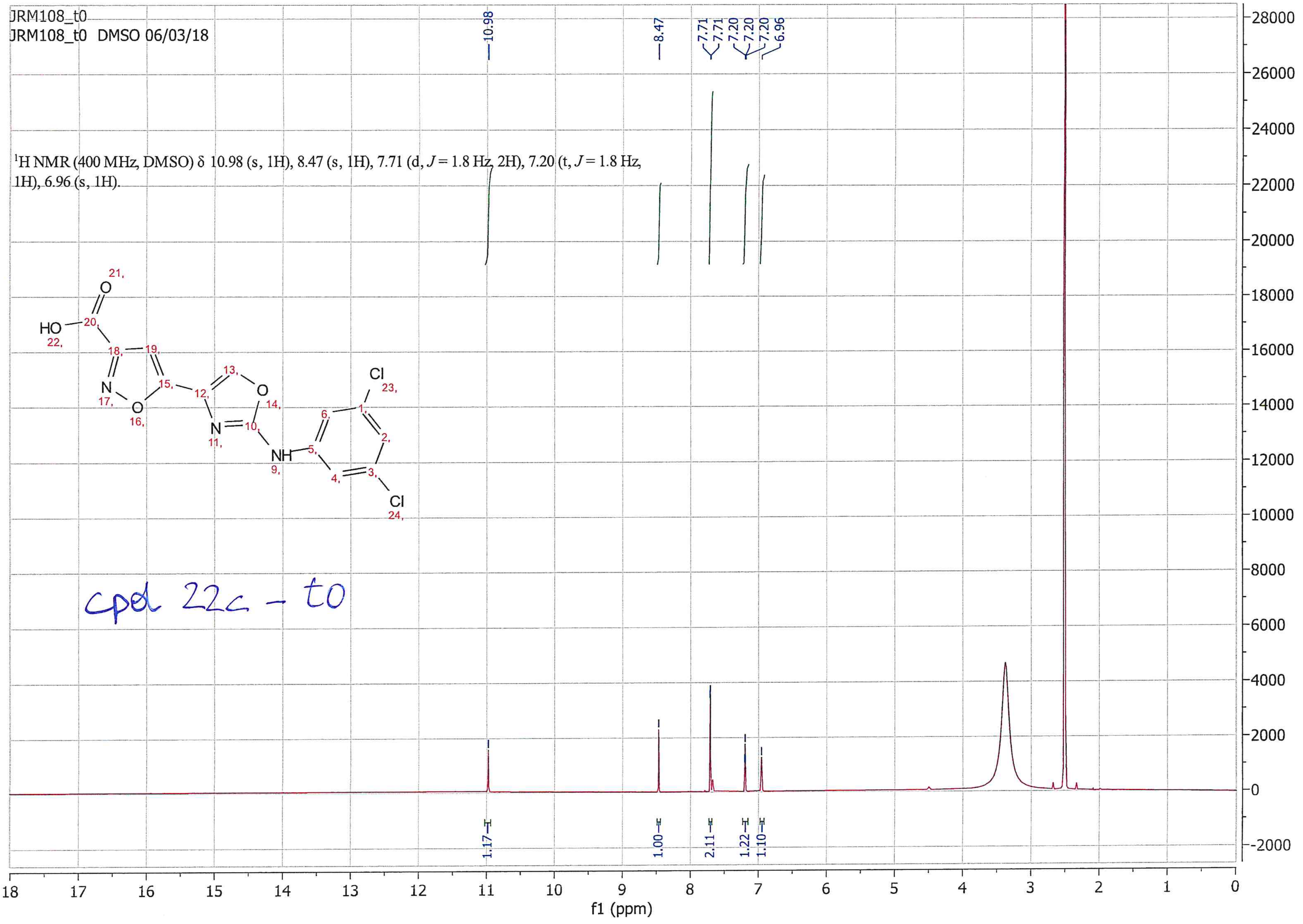
JRM108_t0
JRM108_t0 DMSO 06/03/18
^{1}H NMR (400 MHz, DMSO) δ 10.98 (s, 1H), 8.47 (s, 1H), 7.71 (d, J = 1.8 Hz, 2H), 7.20 (t, J = 1.8 Hz, 1H), 6.96 (s, 1H).
10.98
8.47
7.71
7.71
7.20
7.20
7.20
6.96
1.17
1.00
2.11
1.22
1.10
HO
O
N
O
N
O
NH
Cl
Cl
cpd 22c - t0
f1 (ppm)

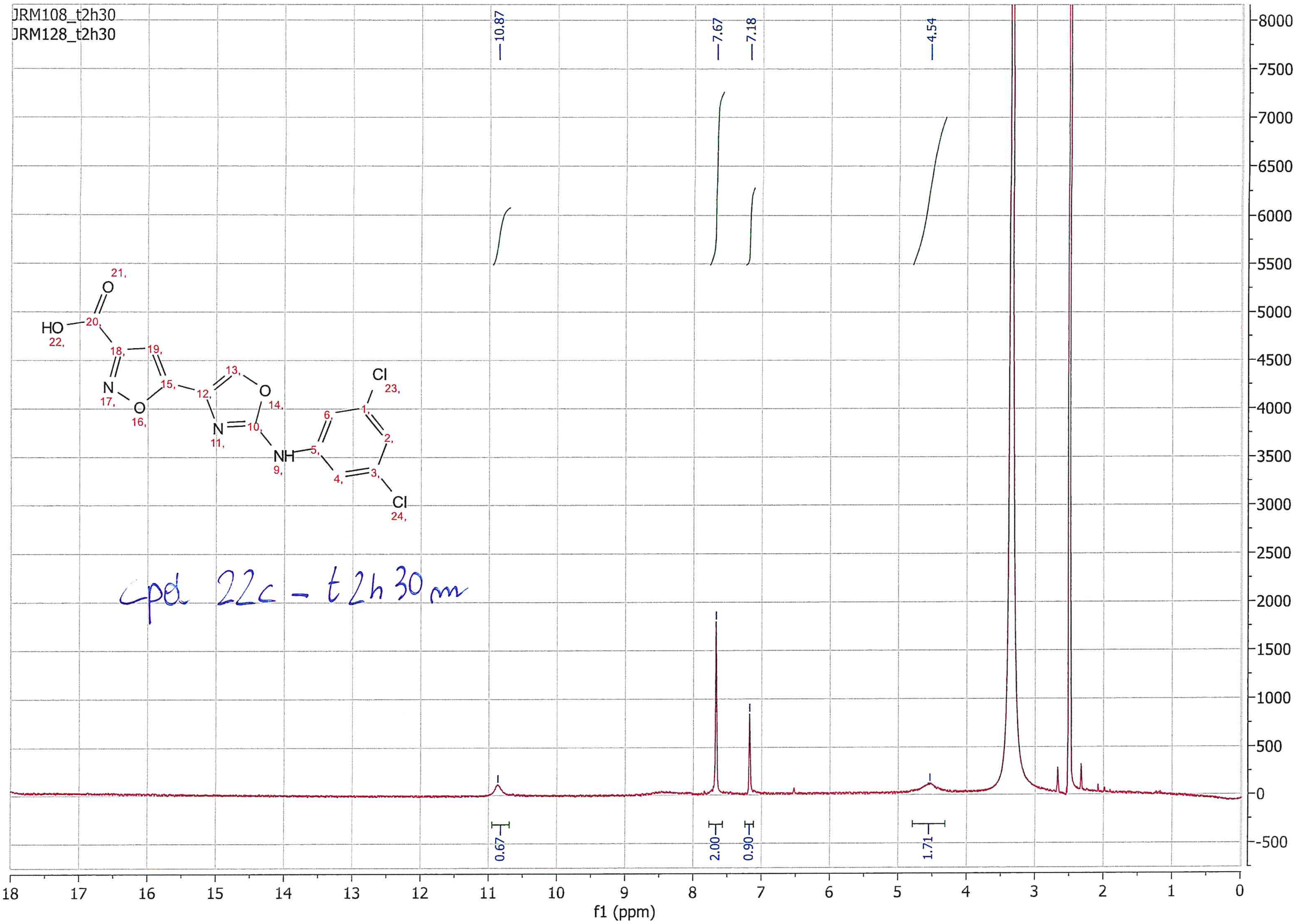

JRM108_t2h30
JRM128_t2h30
10.87
7.67
7.18
4.54
0.67
2.00
0.90
1.71
f1 (ppm)
Cpd 22c - t2h30 mn

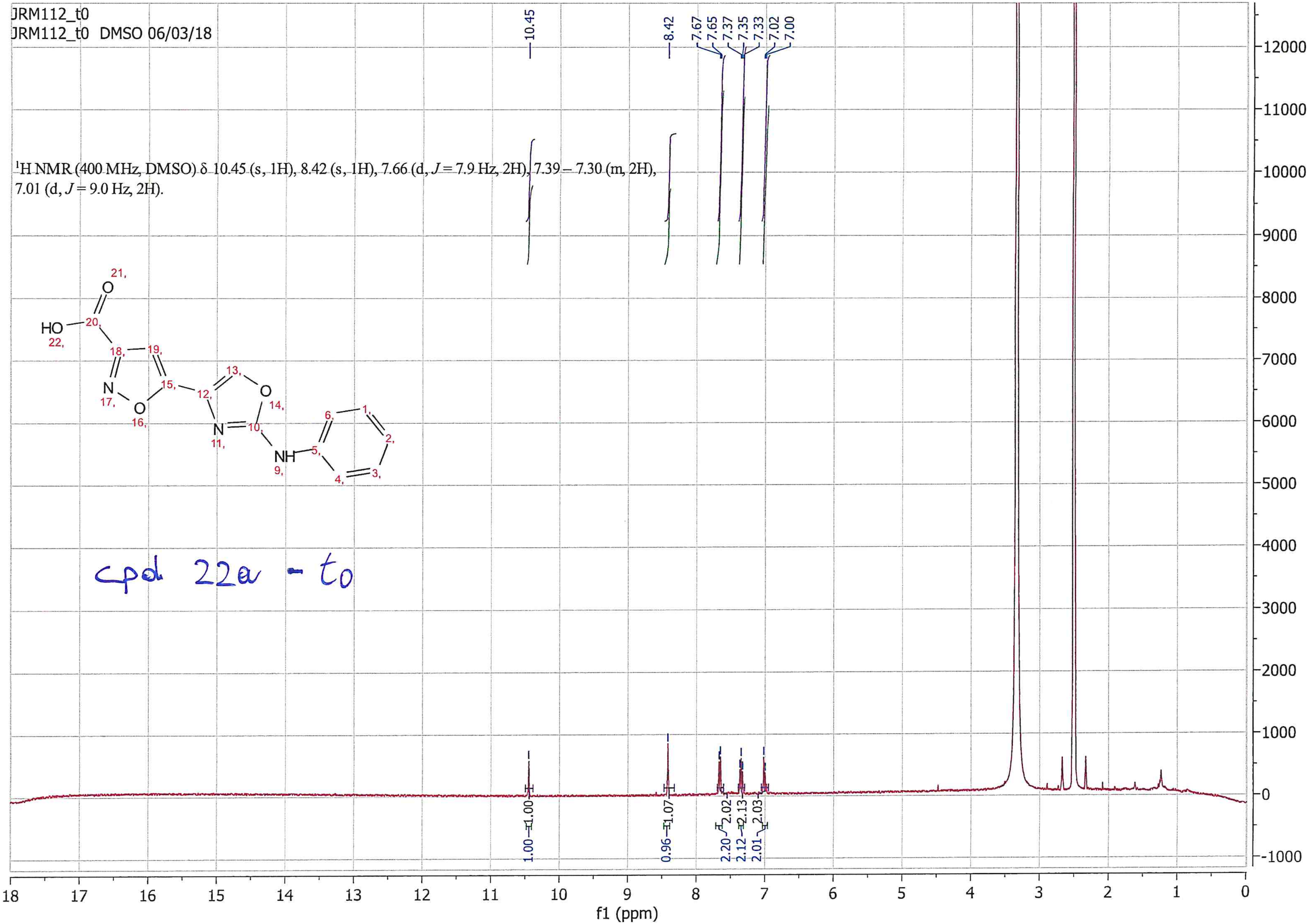
JRM112_t0
JRM112_t0 DMSO 06/03/18
¹H NMR (400 MHz, DMSO) δ 10.45 (s, 1H), 8.42 (s, 1H), 7.66 (d, J = 7.9 Hz, 2H), 7.39 – 7.30 (m, 2H), 7.01 (d, J = 9.0 Hz, 2H).
cpd 22a - t0
10.45
8.42
7.67
7.65
7.37
7.35
7.33
7.02
7.00
1.00
1.00
0.96
1.07
2.20
2.02
2.12
2.13
2.01
2.03
HO
O
N
NH
f1 (ppm)

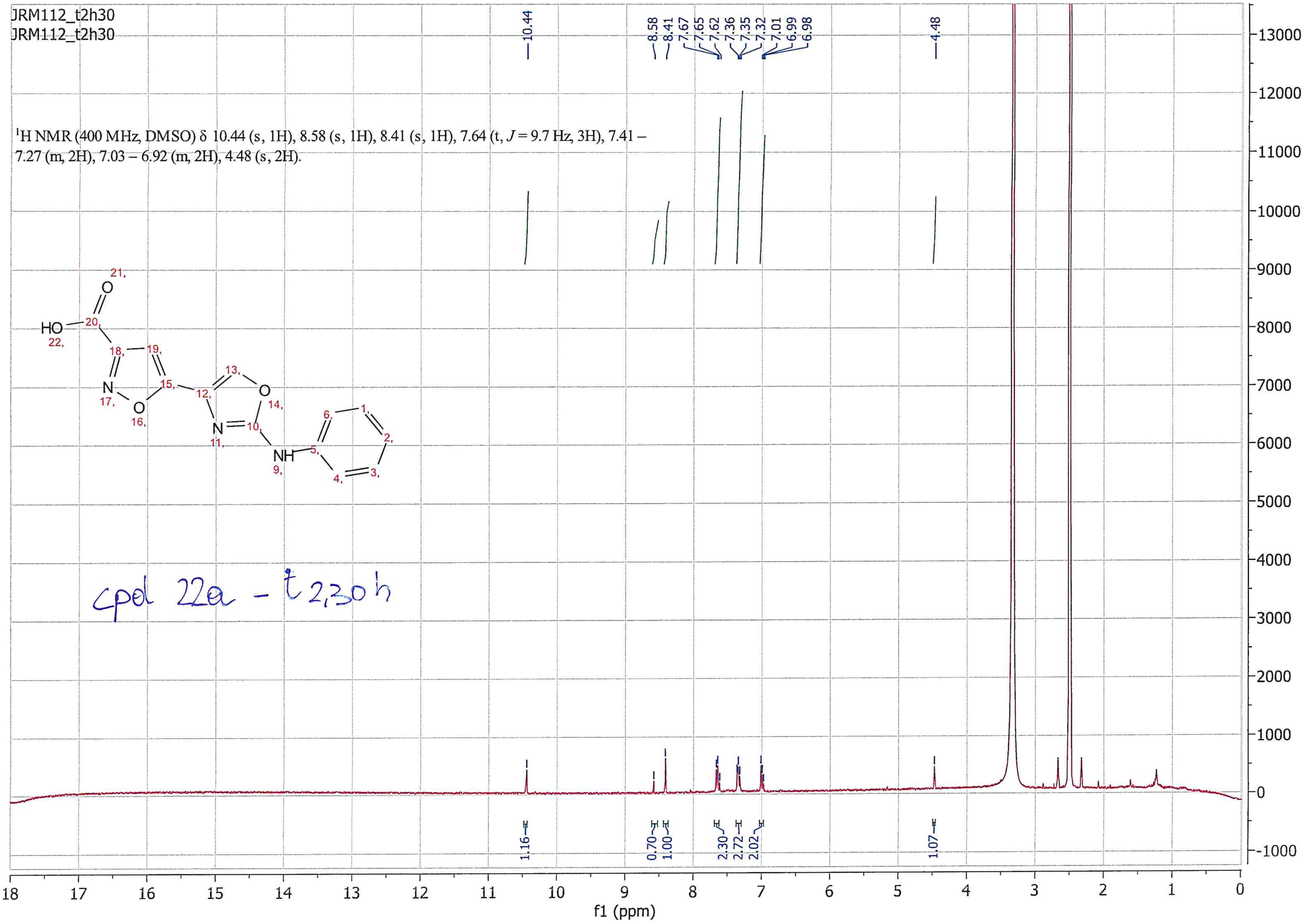
JRM112_t2h30
JRM112_t2h30
10.44
8.58
8.41
7.67
7.65
7.62
7.36
7.35
7.32
7.01
6.99
6.98
4.48
^{1}H NMR (400 MHz, DMSO) δ 10.44 (s, 1H), 8.58 (s, 1H), 8.41 (s, 1H), 7.64 (t, J = 9.7 Hz, 3H), 7.41 – 7.27 (m, 2H), 7.03 – 6.92 (m, 2H), 4.48 (s, 2H).
HO
O
N
O
O
N
NH
cpd 22a - t 2,30 h
1.16
0.70
1.00
2.30
2.72
2.02
1.07
f1 (ppm)

JRM112_t20h30
JRM112_t20h30

^{1}H NMR (400 MHz, DMSO) δ 10.46 (s, 1H), 8.59 (s, 1H), 7.63 (d, J = 8.0 Hz, 2H), 7.41 – 7.28 (m, 2H), 7.00 (t, J = 7.2 Hz, 1H), 4.48 (s, 2H).

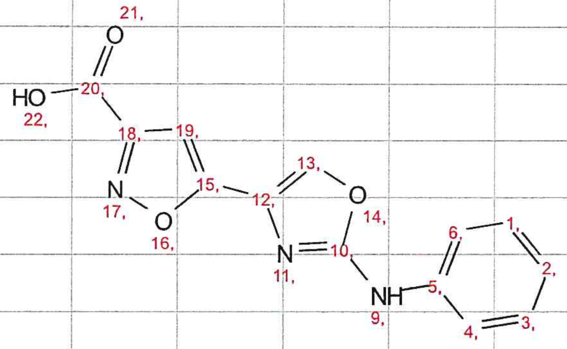

Cpd 22a - t20h30m

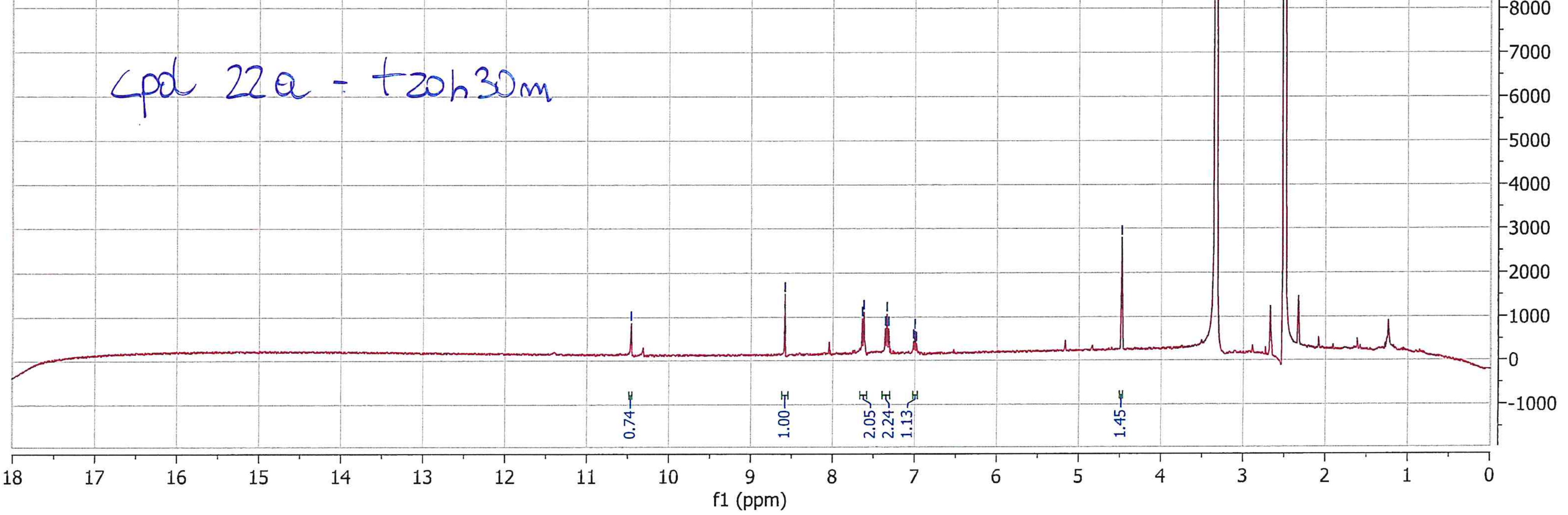

Supplement: Supplementary file 1 [file pharmaceuticals-14-00174-s001.pdf]
